# Cognitive decline in an adult with ATR-16 syndrome due to an unbalanced translocation between 11p15.5 and 16p13.3: a case report

**DOI:** 10.3389/fgene.2025.1595298

**Published:** 2025-05-19

**Authors:** Angela Krutish, Rebekah Kukurudz-Gorowski, Felippe Borlot, Patrick Frosk, Cheryl Rockman-Greenberg, Aizeddin A. Mhanni

**Affiliations:** ^1^ Children’s Hospital Research Institute of Manitoba, University of Manitoba, Winnipeg, MB, Canada; ^2^ Department of Pediatrics and Child Health, University of Manitoba, Winnipeg, MB, Canada; ^3^ Department of Biochemistry and Medical Genetics, University of Manitoba, Winnipeg, MB, Canada; ^4^ Department of Clinical Neurosciences, University of Calgary, Calgary, AB, Canada

**Keywords:** ATR-16 syndrome, case report, chromosome 16p, cognitive decline, dementia

## Abstract

**Background:**

Chromosome 16p13.3 deletions cause a contiguous gene deletion syndrome, ATR-16 syndrome. The classic phenotype of ATR-16 syndrome includes either alpha-thalassemia trait or hemoglobin H disease and intellectual disability; however, considerable variable expressivity has been reported with some patients having only an alpha-thalassemia disorder and others exhibiting a more severe phenotype with additional features.

**Case presentation:**

We describe an adult male with ATR-16 syndrome (due to an unbalanced *de novo* translocation involving chromosomes 11p15.5 and 16p13.3) who developed cognitive decline and increasing dyskinetic movements in his late twenties. Biochemical investigations and exome sequencing did not elucidate an alternative explanation for this decline. Furthermore, neither the deletion on chromosome 16 nor the duplication on chromosome 11 encompassed genes that could explain the decline.

**Conclusion:**

While cognitive decline has not been previously reported in ATR-16 syndrome, this may be another feature of the condition that is subject to variable expressivity. Taking this together with the apparent increased prevalence of dementia in other neurodevelopmental conditions, we hypothesize that individuals with ATR-16 syndrome may be predisposed to early cognitive decline.

## Introduction

ATR-16 syndrome (MIM# 141750) is a contiguous gene deletion syndrome on chromosome 16p13.3 that includes the *HBA1* and *HBA2* loci. The earliest reports of this condition described individuals with hemoglobin H (Hb H) disease, intellectual disability, and congenital anomalies (Rönisch and Kleihauer, 1967; Borochovitz et al., 1970; Weatherall et al., 1981). Restriction enzyme analysis using samples from one of the early patients identified a deletion on chromosome 16 encompassing the alpha-globin gene cluster (Bowcock et al., 1984). Soon after, the responsible locus for this syndrome was more precisely mapped to chromosome 16p13.3 by Giemsa banding (Buckle et al., 1988).

There are several mechanisms through which ATR-16 syndrome can arise. Some cases are *de novo*, while others are inherited from a parent who carries either a balanced translocation or the same deletion as their child (Gibson et al., 2008). As a result of these diverse inheritance mechanisms, some individuals have an isolated deletion on chromosome 16, while others have a second copy number variant elsewhere.

Since the first case report, a multitude of patients with chromosome 16p13.3 deletions and diverse phenotypes have been reported in the literature. The classic phenotype of ATR-16 syndrome is an alpha-thalassemia disorder (either alpha-thalassemia trait or Hb H disease) and mild to moderate intellectual disability (Wilkie et al., 1990); however, a recent report included several families with 16p13.3 deletions and no developmental delay or cognitive disability (Babbs et al., 2020). Other features of ATR-16 syndrome are quite variable and include seizures, craniofacial dysmorphisms, skeletal abnormalities, and genitourinary anomalies (Babbs et al., 2020; MIM, 2008). Though adults have been included in various studies of ATR-16 syndrome, the natural history of this condition in adulthood has not been well documented.

In this report, we describe an adult male with ATR-16 syndrome due to an unbalanced translocation involving chromosomes 16p13.3 and 11p15.5 and cognitive decline beginning in his late twenties.

## Case presentation

The male patient was assessed at age 7 years for a learning disability and dysmorphic features. He was born at 39 weeks gestation by spontaneous vaginal delivery to non-consanguineous parents in their early 30s of Ukrainian and English ancestry. The perinatal history was unremarkable. His birth weight was 3.65 kg (75th%ile). The patient met his early developmental milestones at appropriate ages, but required extra support upon entering elementary school. His physical examination at 7 years revealed a weight of 27 kg (60th%ile), a height of 134 cm (90th%ile), and a head circumference of 53.5 cm (70th%ile). He had dysmorphic features, including mild scaphocephaly, a long and narrow face, high forehead, hypertelorism with downslanting palpebral fissures, micrognathia, a high-arched palate, and prominent ears. His initial work up at that time included a karyotype analysis and fragile X molecular testing, in addition to plasma amino acids, urine amino acids, and urine organic acids (standard of care at the time), all of which were normal.

He was re-evaluated at age 16 years due to tall stature and possible diagnosis of Marfan syndrome. Physical examination revealed a Marfanoid habitus, myopathic facies, long digits, hammer toes and pes planus, mildly hyperextensible and velvety skin, kyphoscoliosis, and pectus carinatum. Electromyography (EMG), nerve conduction studies (NCS), and cardiac evaluations were normal. MRI spine revealed mild atrophy of the cervical, thoracic, and lumbar spinal cord, as well as a syrinx or hydromelia in the distal cord and conus. The patient did not meet the clinical criteria for Marfan syndrome and myotonic dystrophy type 1 molecular testing was normal.

At 30 years, the patient presented with a 2-year history of cognitive decline and increasing dyskinetic movements. This decline included periods of confusion, severely impaired working memory, intermittent urinary incontinence, and irregular sleep. The family also described hand and oral automatisms, episodic behavioural outbursts, and periods of lack of responsiveness. In early adulthood, he was able to work part-time, volunteer, drive, and socialize; however, by 30 years he was no longer able to do these activities. Behavioural and psychological interventions had little impact; various psychiatric medications were trialled but discontinued due to lack of efficacy or side effects.

Multiple electroencephalogram (EEG) studies were performed in wakefulness and sleep, none of which showed epileptiform discharges or focal abnormalities. Brain MRI at the onset of his cognitive decline at 29 years was unremarkable. Brain MRI at 33 years revealed a 3 mm by 4 mm pituitary microadenoma that was non-functional. Follow-up MRIs at 34 years revealed two ischemic lesions (a chronic cortical infarct in the left parietal cortex and a lacunar infarct in the inferior right cerebellar nucleus), and a third lesion was observed in the inferior right cerebellar nucleus at 35 years. Echocardiogram subsequently demonstrated preserved ejection fraction with a hypermobile septum and a patent foramen ovale, which is thought to be related to the ischemic lesions. An autoimmune encephalitis panel, hypercoagulable and antiphospholipid antibody work-up, vasculitis work-up, CT angiography of the Circle of Willis, and Holter monitoring were all normal.

Biochemical investigations looking for an inborn error of metabolism were all non-diagnostic. Chromosomal microarray analysis and fluorescence *in situ* hybridization (FISH) studies revealed an unbalanced translocation: ish der(16)t(11; 16)(p15.5; p13.3); parental FISH studies were normal, suggesting that the translocation was *de novo*. The 11p15.5 duplication of uncertain significance spanned 1.1 Mb and did not overlap with the imprinting control regions for Silver-Russell syndrome or Beckwith-Wiedemann syndrome. The 16p13.3 pathogenic deletion spanned 1.6 Mb and encompassed the region associated with ATR-16 syndrome. Tables 1, 2 list the MIM-disease associated genes encompassed by the patient’s duplication and deletion, respectively. The patient had consistently low mean corpuscular volume (MCV) (in the range of 67–70 fL; normal: 80–90 fL) as expected in ATR-16 syndrome. Given the lack of explanation for the patient’s decline, exome sequencing (ES) was performed (SickKids Genome Diagnostics Laboratory; Toronto, ON). ES identified variants in two autosomal recessive genes: (1) a heterozygous pathogenic variant in *HEXA* (NM_000520.6: c.805G>A, p.Gly269Ser), and (2) a heterozygous pathogenic variant in *ACSF3* (NM_174917.5: c.1672C>T, p.Arg558Trp). Enzymatic analyses of hexosaminidase A and total hexosaminidase were consistent with carrier status for Tay-Sachs disease. Urine organic acids were normal, consistent with carrier status for combined malonic and methylmalonic aciduria.

**TABLE 1:** Disease genes encompassed by the patient’s 11p15.5 duplication.

Gene	Chromosome location^a^	MIM#: Phenotype^b^
*IFITM5*	chr11:298,200-299,526	610967: Osteogenesis imperfecta type V (AD)
*IFITM3*	chr11:319,676-320,860	614680: Severe influenza virus infection^c^
*RNH1*	chr11:494,515-507,242	620461: Infection-induced acute encephalopathy-12^c^ (AR)
*HRAS*	chr11:532,242-535,576	218040: Costello syndrome (AD) & Congenital myopathy with excess of muscle spindles (AD)
*LRRC56*	chr11:537,527-554,912	618254: Primary ciliary dyskinesia-39 (AR)
*IRF7*	chr11:612,555-615,950	616345: Immunodeficiency-39^d^ (AR)
*DRD4*	chr11:637,269-640,706	143465: Attention deficit-hyperactivity disorder^c^ (AD)
*DEAF1*	chr11:644,220-707,083	615828: Vulto-van Silfout-de Vries syndrome (AD) 617171: Neurodevelopmental disorder with hypotonia, impaired expressive language, and with or without seizures (AR)
*EPS8L2*	chr11:706,231-727,727	617637: Deafness-106 (AR)
*TALDO1*	chr11:747,464-765,012t	606003: Transaldolase deficiency (AR)
*SLC25A22*	chr11:790,475-798,281	609304: Developmental and epileptic encephalopathy-3 (AR)
*PIDD1*	chr11:799,184-805,231	619827: Intellectual developmental disorder-75 with neuropsychiatric features and variant lissencephaly (AR)
*PNPLA2*	chr11:818,914-825,573	610717: Neutral lipid storage disease with myopathy (AR)
*CD151*	chr11:832,952-838,831	609057: Epidermolysis bullosa simplex-7 with nephropathy and deafness (AR)

^a^ Chromosome location is based on GRCh37 (hg19).

^b^ (AD) and (AR) indicate phenotypes that follow autosomal dominant and autosomal recessive patterns of inheritance, respectively.

^c^ Indicates susceptibility to the phenotype.

^d^ Indicates a provisional relationship between the gene and phenotype.

**TABLE 2:** Disease genes encompassed by the patient’s 16p13.3 deletion.

Gene	Chromosome location^a^	MIM#: Phenotype^b^
*NPRL3*	chr16:135,385-188,672	617118: Familial focal epilepsy with variable foci-3 (AD)
*HBA2*	chr16:222,875-223,709	617981: Familial erythrocytosis-7 (AD) 140700: Heinz body anemia (AD) 613978: Hemoglobin H disease 604131: Alpha-thalassemia
*HBA1*	chr16:226,679-227,521	617981: Familial erythrocytosis-7 (AD) 140700: Heinz body anemia (AD) 613978: Hemoglobin H disease 617973: Methemoglobinemia (AD) 604131: Alpha-thalassemia
*STUB1*	chr16:730,410-732,801	618093: Spinocerebellar ataxia-48 (AD) 615768: Spinocerebellar ataxia-16 (AR)
*CCDC78*	chr16:772,582-776,880	614807: Centronuclear myopathy-4^c^ (AD)
*CACNA1H*	chr16:1,203,106-1,271,768	611942: Childhood absence epilepsy-6^d^ 611942: Idiopathic generalized epilepsy-6^d^ 617027: Familial hyperaldosteronism type IV (AD)
*CLCN7*	chr16:1,494,936-1,525,029	618541: Hypopigmentation, organomegaly, and delayed myelination and development (AD) 166600: Osteopetrosis-2 (AD) 611490: Osteopetrosis-4 (AR)

^a^ Chromosome location is based on GRCh37 (hg19).

^b^ (AD) and (AR) indicate phenotypes that follow autosomal dominant and autosomal recessive patterns of inheritance, respectively.

^c^ Indicates a provisional relationship between the gene and phenotype.

^d^ Indicates susceptibility to the phenotype.

## Discussion

Here we describe an adult male with ATR-16 syndrome due to a *de novo* unbalanced translocation involving chromosomes 11p15.5 and 16p13.3. Many of the patient’s clinical features (i.e., intellectual disability, craniofacial dysmorphisms, and low MCV) are in keeping with the known presentation of ATR-16 syndrome. However, the patient developed cognitive decline near the end of his third decade of life, which has not been previously described. While the patient was noted to have three small, asymptomatic intracranial lesions, these developed 5 years after the onset of his cognitive decline. The lack of chronology between the appearance of these lesions and the cognitive decline argues that these are not the cause of his decline. Given that an exhaustive work-up has not revealed alternative explanations, we hypothesize that individuals with ATR-16 syndrome may be at increased risk for early cognitive decline.

We considered whether the patient’s duplication or deletion may encompass a single gene that could explain his early cognitive decline. None of the 14 disease genes encompassed by the 11p15.5 duplication are known to be individually associated with cognitive changes in adulthood. Of the seven disease genes encompassed by the 16p13.3 deletion, *STUB1*, associated with autosomal dominant spinocerebellar ataxia-48 (SCA48, MIM, 2022b) and autosomal recessive spinocerebellar ataxia-16 (SCA16, MIM, 2022a), is the only gene that overlaps with the patient’s history of decline. However, the disease mechanism in SCA48 is not well understood. In fact, a digenic pattern of inheritance has been explored (Barbier et al., 2023). Furthermore, the majority of pathogenic *STUB1* variants are missense variants and only one publication has suggested haploinsufficiency as a mechanism of disease in SCA48 (Roux et al., 2020). We also noted that the patient has neither characteristic brain findings of SCA48 (i.e., cerebellar or cortical atrophy, T2-weighted hyperintensities in the cerebellar dentate nuclei) nor gait ataxia at this time. Finally, because many of the 16p13.3 deletions reported in the literature encompass the *STUB1* gene, we expect that this phenotype would have been reported previously if it simply is a product of heterozygous *STUB1* deletion.

There appears to be a significant degree of variable expressivity in ATR-16 syndrome. This is demonstrated by a cohort study of 41 individuals from 27 families with pure 16p13.3 monosomy; among the 41 individuals, 56% had alpha-thalassemia trait only, 24% had alpha-thalassemia trait and at least one other feature without skeletal anomalies, and 20% had alpha-thalassemia trait, developmental delay/speech delay/intellectual disability, dysmorphic features, and skeletal anomalies (Babbs et al., 2020). This finding is not exclusive to ATR-16 syndrome, given that many single gene conditions and deletion syndromes exhibit variable expressivity and reduced penetrance (Deak et al., 2011; Goh et al., 2025); however, the mechanisms underlying phenotypic expression are often unclear. In general, Babbs et al. (2020) observed that individuals with more severe phenotypes tended to have larger 16p13.3 deletions (greater than 1 Mb); however, neither the size of the deletion nor the genes encompassed by the deletion was sufficient to distinguish individuals in the cohort with intellectual disability from those without. Our patient aligns with these observations, given that he carries a 1.6 Mb deletion and his phenotype includes alpha-thalassemia trait, intellectual disability, dysmorphic features, and skeletal anomalies. Although cognitive decline has not been previously reported in ATR-16 syndrome, the pervasiveness of variable expressivity in this syndrome and other genetic conditions, combined with the patient’s relatively large deletion, suggest that premature cognitive decline could plausibly be part of the natural history of ATR-16 syndrome.

Multiple mechanisms underlying the variable expressivity of intellectual disability in ATR-16 syndrome have been proposed and were considered as possible mechanisms for our patient’s phenotype. For example, the presence of a second copy number variant (which is common in individuals with ATR-16 syndrome) was postulated to modulate the phenotype (Wilkie et al., 1990). While our patient does carry a substantial duplication on chromosome 11p15.5, duplication of this region is not associated with any known syndromes and it does not involve the imprinting control regions for Silver-Russell or Beckwith-Wiedemann syndromes; as such, the chromosome 11 duplication is not felt to be the cause of this patient’s early cognitive decline. Babbs et al. (2020) proposed that modifying loci elsewhere in the genome may contribute to the ATR-16 phenotype. This was based on their observation of coinheritance of a 16p13.3 deletion and a variant in a relevant disease gene (specifically *SMAD6* and *NRXN1*) in two families. ES for our patient did not identify any pathogenic variants in other disease genes that would explain the cognitive decline. While common variants could still be contributing to his phenotype, the effects of each common variant would likely be small; for example, in a large cohort study of patients with 22q11.2 deletion syndrome, polygenic risk scores for IQ explained only 3.8% of the variance in IQ (Davies et al., 2020). Finally, while Babbs et al. (2020) proposed other mechanisms for the variable expressivity underlying ATR-16 syndrome, including compensatory gene expression and telomere position effects, their experimental investigations did not corroborate these mechanisms. In summary, we did not find evidence of any reasonable alternative genomic explanations for the progression in our patient’s phenotype.

Notably, premature cognitive decline has been reported in other neurodevelopmental conditions. A classic example is trisomy 21, in which there is an increased risk of Alzheimer disease and a greater than 90% lifetime risk of dementia (Fortea et al., 2021). 22q11.2 deletion syndrome (especially in individuals who later develop a psychotic disorder), Prader-Willi syndrome, and autism are other examples in which cognitive decline has been reported (Sinnema et al., 2012; Vorstman et al., 2015; Klein et al., 2023). Furthermore, population studies in Canada, the United Kingdom, and Japan reported increased prevalence and earlier age of onset of dementia in individuals with intellectual and developmental disabilities (Strydom et al., 2010; Shooshtari et al., 2011; Takenoshita et al., 2020). This has been attributed to lower cognitive reserve, which renders the brain less resistant to neuropathology leading to dementia (Sheerin et al., 2020; Livingston et al., 2024). Increased access to genetic testing means that more adults with intellectual disability are known to have an underlying genetic condition. Furthermore, improvements in pediatric care for individuals with genetic conditions has led to increasing survival rates well into adulthood (Malecki et al., 2024). As such, baseline assessments of cognitive function in early adulthood and longitudinal monitoring are needed to better understand the cognitive trajectories and aging processes in neurodevelopmental conditions (Kwetsie et al., 2024), as well as to provide anticipatory guidance to families and ensure appropriate care is available.

Given the considerable degree of variable expressivity in individuals with ATR-16 syndrome and the apparent increased prevalence and decreased age of onset for dementia in neurodevelopmental conditions, we postulate that individuals with ATR-16 syndrome may be predisposed to early cognitive decline. Our report potentially expands what is known about the natural history and the neurodevelopmental trajectory of ATR-16 syndrome. We highlight this phenomenon in ATR-16 syndrome to alert clinicians and researchers of the importance of monitoring their patients for decline in cognitive function, similar to that observed in our patient. Identifying additional individuals with ATR-16 syndrome and cognitive decline would provide an opportunity for a cohort study to better understand the mechanisms underlying variable expressivity in this condition, as well as in other neurodevelopmental disorders.

## Data Availability

The original contributions presented in the study are included in the article/supplementary material, further inquiries can be directed to the corresponding author.
